# The contribution of HPV18 to cervical cancer is underestimated using high-grade CIN as a measure of screening efficiency

**DOI:** 10.1038/sj.bjc.6603693

**Published:** 2007-03-20

**Authors:** S Bulk, J Berkhof, L Rozendaal, N C Fransen Daalmeijer, M Gök, F A de Schipper, F J van Kemenade, P J F Snijders, C J L M Meijer

**Affiliations:** 1 Department of Pathology, VU University Medical Center, PO Box 7057, 1007 MB, Amsterdam, The Netherlands; 2 Department of Clinical Epidemiology and Biostatistics, VU University Medical Center, PO Box 7057, 1007 MB, Amsterdam, The Netherlands; 3 Department of Obstetrics and Gynecology, Hospital Walcheren, PO Box 3200, 4380 DD, Vlissingen, The Netherlands

**Keywords:** cervical intraepithelial neoplasia, squamous cell carcinoma, human papillomavirus, epidemiology, PALGA

## Abstract

In one geographical area, 14 high-risk human papillomavirus types in cervical intraepithelial neoplasia (CIN2/3; *n*=139) and cervical squamous cell carcinoma (SCC; *n*=84) were analysed. HPV18 was more prevalent in SCC than CIN2/3 (OR 9.8; 95% confidence interval: 2.5–39). Other high-risk types prevalences corresponded in CIN2/3 and SCC. Evaluations using CIN2/3 as a measure of efficiency underestimate the contribution of HPV18 to SCC.

Human papillomavirus (HPV) infections are the cause of cervical cancer and its precursors lesions (Walboomers *et al*, 1999). So far, 15 HPV types have been classified as high-risk types, and three types have been classified as probable high risk (Munoz *et al*, 2003). Within the group of high-risk HPV (hrHPV) types, different types seem to confer different degrees of risk for cervical cancer and its precursor lesions (Clifford *et al*, 2006). A markedly increased risk of cervical cancer has been attributed to HPV16 and HPV18, as prevalence studies have shown that 60–70% of cervical squamous cell carcinoma (SCC) cases are positive for HPV16 or HPV18 (Munoz *et al*, 2003).

As a consequence, assessment of the additive value of HPV typing in cervical screening is the subject of many ongoing studies. However, screening evaluations generally use high-grade cervical intraepithelial neoplasia (CIN2/3) as intermediate end point of cervical cancer. This may lead to underestimation or overestimation of the contribution of certain HPV types in case the HPV type distribution between CIN2/3 and cervical cancer differs. Indeed, several studies suggest that HPV18 is underrepresented in CIN2/3 compared to SCC (Bulkmans *et al*, 2005; Castle *et al*, 2005; Khan *et al*, 2005), and in a meta-analysis as well (Clifford *et al*, 2006).

Here, we address type distribution of different hrHPV types in CIN2/3 and SCC samples collected in one geographical area.

## MATERIALS AND METHODS

### Study selection

From the archives of the Department of pathology of Ziekenhuis Walcheren in The Netherlands, we identified 187 specimens of CIN2/3 (87 CIN2 and 100 CIN3) diagnosed after screening between 1996 and 2000, and 90 specimens of SCC from the period 1981 to 1998. The department of pathology serves the population of the Walcheren peninsula, where follow-up of screened women is approximately 90% complete. All histological samples were collected retrospectively and were subjected to revision. The Medical Ethics Committee of VU University Medical Center approved the study (2001/179).

### HPV testing

Representative samples of the formalin-fixed biopsies were processed for PCR analysis (Walboomers *et al*, 1999; Jacobs *et al*, 2000). Amplification of a 209 base-pair fragment of the *β*-globin gene was performed to test the integrity of DNA (Roda Husman *et al*, 1994). Detection of hrHPV was performed by GP 5+/6+ consensus primer PCR enzyme immunoassay, using a cocktail of 21 low-risk types and 14 high-risk types. All HPV-positive samples were typed by reverse line blotting (van den Brule *et al*, 2002). We tested for hrHPV16, 18, 31, 33, 35, 39, 45, 51, 52, 56, 58, 59, 66, and 68 and lrHPV6, 11, 32, 40, 42, 43, 44, 54, 55, 57, 61, 70, 71, 72, 81, 83, 84, 85, 86, cp6108 and jc9710 (Jacobs *et al*, 1997). In addition, all GP5+/6+ PCR-negative samples were evaluated by E7 region type-specific PCR for the same 14 high-risk types to exclude the presence of hrHPV in integrated form and/or loss of the GP5+/6+ primer-binding region (Walboomers *et al*, 1999). Technicians performing PCR analysis were blinded for histological classification of samples.

### Study material

Formalin-fixed archival material could not be retrieved for 17 cases with CIN2/3. Of the women with CIN2/3, 21 samples tested HPV-negative and in seven cases, only low-risk types could be identified. One case tested hrHPV-positive without identifying a specific high-risk type, leaving 139 cases of CIN2/3 with known hrHPV type for statistical analysis. Of the women with SCC, seven cases tested negative for HPV by both assays, leaving 83 cases with SCC and a known hrHPV type for statistical analysis.

### Statistical analysis

Differences in prevalence of hrHPV types for CIN2/3 compared to SCC were examined using the Mantel-Haenszel common odds ratio (OR_MH_) and 95% confidence intervals (95% CIs). Statistical significance was tested by Cochran's Mantel-Haenszel test. Data were stratified by age (i.e., below 29 years, 29–33, 34–38, 39–43, 44–48, 49–53, 54–58, 59–61 and 62 years and over) and whether infections were single or multiple. The presence of an association between OR_MH_ and age was tested by Breslow–Day's test of homogeneity. Analyses were repeated for women with single infections only.

The presented results may not be free of hidden bias as there is some evidence that HPV18-positive lesions are underrated by cytology (Woodman *et al*, 2003; Berkhof *et al*, 2006). Therefore, it cannot be excluded that prevalence differences of certain hrHPV types in CIN2/3 compared to SCC is associated with cytological screening failures. We examined the influence of hidden bias by computing an adjusted *P*-value obtained under the assumption that among the hrHPV-positive CIN2/3 and SCC cases, the odds of observing a certain hrHPV type in SCC compared to CIN2/3 differed by a factor 2 (Rosenbaum (1991).

## RESULTS

The mean age of women with CIN2/3 (*n*=139) was 37 years (range 26–57 years). Women with SCC (*n*=84) had a mean age of 49 years (range 27–88 years). The mean age of women with SCC was higher than the mean age of women with CIN2/3 (*P*<0.001). Multiple HPV infections were present in 9.4% (13 out of 139) of CIN2/3, and in 10.8% (9 out of 83) of SCC (*P*=0.719).

Table 1 displays the hrHPV type-specific prevalence rates in women with CIN2/3 compared to SCC. In women with CIN2/3, 59.7% of hrHPV positivity was due to HPV16 and 12.9% to HPV31. Another 2.9% of hrHPV infections were HPV18 and 11.5% HPV52 positive. In women with SCC, 69.9% of hrHPV positivity was due to HPV16. HPV31 was present in 7.2% of cases, and HPV18 in 12.0%. No SCC cases tested positive for HPV52 in this study. Type-specific prevalences did not substantially change when analysing women with single infections separately.

Women having SCC carried HPV16 more often than women with CIN2/3, although the estimated OR_MH_ was not statistically significant (OR_MH_ 1.5; 95% CI: 0.8–3.1). The prevalence of HPV18 was significantly higher in SCC than in CIN2/3 (OR_MH_ 8.3; 95% CI: 1.9–36). The other high-risk HPV types were not associated with increased risks individually, but when all hrHPV-positive infections without HPV16 and HPV18 were analysed as one group, the result was significant (OR_MH_ 0.4; 95% CI: 0.2–0.8). For none of the HPV types OR varied with age (data not shown). Results for single infections were comparable.

Even when assuming that the odds to detect an HPV18-associated SCC compared to an HPV18-associated CIN2/3 differed by a factor 2, HPV18 would still be increased in SCC compared to CIN2/3 (*P*=0.035).

## DISCUSSION

In this cross-sectional study, we compared hrHPV type-specific prevalence in CIN2/3 and invasive carcinomas in women identified through population-based cervical screening in a geographically restricted area. We demonstrated that HPV18 prevalence is increased in SCC compared to premalignant lesions. In addition, this study underlines the previously recognised importance of HPV16 for the development of SCC. Other types did not show a prevalence difference.

The increased prevalence of HPV18 in cervical cancer compared to CIN2/3 as found in our study is in agreement with a recent meta-analysis and data from Australia, South Africa and the US (Clifford *et al*, 2003; Kay *et al*, 2003; Zuna *et al*, 2004). The sample size of our study was slightly low, because CIN2/3 and SCC cases were collected from one restricted geographical area. Nonetheless, we were able to demonstrate effects of HPV18 in SCC. Our study cannot differentiate between increased progression risks of HPV18 for the transition of CIN2/3 to SCC as an explanation of our findings, and underdetection of screen-diagnosed CIN2/3 lesions associated with HPV18 as an alternative theory. Our previous, cross-sectional study indicated a preferential risk of HPV18 for the development of cancer from normal cytology (Bulk *et al*, 2006). However, the estimate of the risk associated with HPV18 for cervical cancer compared to CIN2/3 obtained in this study is rather high (OR_MH_ 9.7; 95% CI: 2.4–39). To calculate approximately how robust this finding is against underdetection of HPV18-associated lesions by cytology in a cervical screening programme (Woodman *et al*, 2003), we estimated the prevalence difference of HPV18 between CIN2/3 and SCC using a sensitivity analysis. We found that the difference in HPV18 prevalence between CIN2/3 and SCC still remains statistically significant, even if the detection of HPV18-associated CIN2/3 lesions through screening compared to HPV18-positive SCC cases is underestimated by a factor 2. Underrepresentation of HPV18-associated lesions might be explained by the finding that HPV18 infections often occur high in the endocervical canal where lesions are less accessible to screening. Nevertheless, whether the observed association between HPV18 and SCC is mainly caused by a preferential risk of HPV18 for the development if SCC, or by underdetection of HPV18 precursor lesions, our findings have important implications for evaluations of screening programmes. Currently, most, if not all, screening programmes use CIN2/3 as an intermediate end point for cervical cancer to evaluate screening efficacy. This approach seriously underestimates the contribution of HPV18 to the development of SCC. As a consequence, vaccination to HPV16 and HPV18 will decrease HPV18-associated carcinoma incidence more than short-term studies using CIN2/3 as an outcome measure indicate.

In conclusion, HPV18 prevalence is high in invasive SCC compared to CIN2/3 lesions. Thus, when CIN2/3 is used as intermediate end point for cervical cancer, the contribution of HPV18 to the development of invasive SCC is underestimated. As the vast majority of both SCC and adenocarcinomas is attributable to HPV16 and HPV18, women with screen-detected HPV16 and HPV18 infections should be offered more intensive follow-up schemes compared to women infected with other high-risk HPV types.

## Figures and Tables

**Table 1:** HrHPV type-specific prevalence rates in high-grade cervical intraepithelial neoplasia *vs* squamous cell carcinoma

	**All infections** a ,b				**Single infections** a			
	**SCC**	**CIN2/3**	**SCC∼>CIN2/3**		**SCC**	**CIN2/3**	**SCC∼>CIN2/3**	
	***n*=84**	***n*=139**			***n*=74**	***n*=126**		
**Type**	***N* (%)**	***N* (%)**	**OR (95% CI)**	***P***	***N* (%)**	***N* (%)**	**OR (95% CI)**	***P***
16	58 (69.9)	83 (59.7)	1.5 (0.8–3.1)	0.229	55 (74.3)	75 (59.5)	2.1 (1.0–4.5)	0.065
18	**10 (12.0)**	**4 (2.9)**	**8.3 (1.9–36)**	**0.002**	**8 (10.8)**	**3 (2.4)**	**10 (2.0–52)**	**0.005**
								
n16/n18	**17 (20.5)**	**51 (36.7)**	**0.4 (0.2–0.8)**	**0.009**	**11 (14.9)**	**47 (37.3)**	**0.2 (0.1–0.5)**	**0.001**
31	6 (7.2)	18 (12.9)	0.4 (0.1–1.5)	0.145	3 (4.1)	11 (8.7)	0.5 (0.1–2.1)	0.371
33	3 (3.6)	8 (5.8)	1.0 (0.2–4.6)	0.959	1 (1.4)	7 (5.6)	0.4 (0.1–3.4)	0.390
35	2 (2.4)	5 (3.6)	0.2 (0.1–3.3)	0.272	—	3 (2.4)	—	—
39	4 (4.8)	—	—	—	1 (1.4)	—	—	—
45	5 (6.0)	6 (4.3)	0.8 (0.1–6.1)	0.850	4 (5.4)	3 (2.4)	0.6 (0.1–8.1)	0.701
51	—	4 (2.9)	—	—	—	3 (2.4)	—	—
52	—	16 (11.5)	—	—	—	14 (11.1)	—	—
56	2 (2.4)	1 (0.7)	1.6 (0.1–46)	0.815	1 (1.4)	1 (0.8)	1.7 (0.1–28)	0.705
58	2 (2.4)	7 (5.0)	1.6 (0.1–11)	0.652	1 (1.4)	4 (3.2)	0.6 (0.1–9.0)	0.738
59	—	1 (0.7)	—	—	—	1 (0.8)	—	—
66	—	1 (0.7)	—	—	—	—	—	—
68	—	—	—	—	—	—	—	—

CIN2/3=cervical intraepithelial neoplasia; HrHPV=high-risk human papillomavirus; SCC, squamous cell carcinoma.

Bold typeface indicates *P*<0.05.

a Analyses are adjusted for age in 5-year strata.

b Multiple and single infections combined. Analyses are adjusted for multiplicity of infection.
